# Time Coherent Full-Body Poses Estimated Using Only Five Inertial Sensors: Deep versus Shallow Learning

**DOI:** 10.3390/s19173716

**Published:** 2019-08-27

**Authors:** Frank J. Wouda, Matteo Giuberti, Nina Rudigkeit, Bert-Jan F. van Beijnum, Mannes Poel, Peter H. Veltink

**Affiliations:** 1 Department of Biomedical Signals & Systems, Technical Medical Centre, University of Twente, P.O. Box 217, 7500 AE Enschede, The Netherlands; 2 RADiCAL Solutions, LLC. 125 West 31st Street, New York, NY 10001, USA; 3 Xsens Technologies B.V., Pantheon 6a, 7521 PR Enschede, The Netherlands; 4 Department of Computer Science, Faculty of Electrical Engineering, Mathematics & Computer Science, University of Twente, P.O. Box 217, 7500 AE Enschede, The Netherlands

**Keywords:** inertial motion capture, machine learning, neural networks, deep learning, LSTM, time coherence, human movement, reduced sensor set, pose estimation

## Abstract

Full-body motion capture typically requires sensors/markers to be placed on each rigid body segment, which results in long setup times and is obtrusive. The number of sensors/markers can be reduced using deep learning or offline methods. However, this requires large training datasets and/or sufficient computational resources. Therefore, we investigate the following research question: “What is the performance of a shallow approach, compared to a deep learning one, for estimating time coherent full-body poses using only five inertial sensors?”. We propose to incorporate past/future inertial sensor information into a stacked input vector, which is fed to a shallow neural network for estimating full-body poses. Shallow and deep learning approaches are compared using the same input vector configurations. Additionally, the inclusion of acceleration input is evaluated. The results show that a shallow learning approach can estimate full-body poses with a similar accuracy (~6 cm) to that of a deep learning approach (~7 cm). However, the jerk errors are smaller using the deep learning approach, which can be the effect of explicit recurrent modelling. Furthermore, it is shown that the delay using a shallow learning approach (72 ms) is smaller than that of a deep learning approach (117 ms).

## 1. Introduction

Capturing full-body human motion can be valuable for various applications, such as biomechanical analysis, virtual/augmented reality, and gaming. For example, the increased use of wearable motion caption systems is helping coaches/athletes to improve their training programs [1]. Patients can benefit from biomechanical analyses to monitor treatment effectiveness [2]. Motion capture also has the potential to estimate kinetic quantities for various activities [3,4,5]. Virtual/augmented reality can produce realistic training environments for patients by providing interaction with the virtual elements using motion capture (e.g., knee osteoarthritis [6] or phantom limb pain [7]). The success of Microsoft Kinect shows that motion capture can also be applied to (serious) gaming (e.g., for traumatic brain injury patients [8] and neurological rehabilitation [9]). Full-body motion capture is currently done by using either body-worn sensors (e.g., inertial measurement units (IMUs) [10]) or external measurement equipment (e.g., cameras [11,12]). These systems typically require users to wear sensors/markers on each (rigid) body segment, e.g., 17 sensors for Xsens MVN [13] and 37 markers for the Plug-In Gait protocol of Vicon [11,14]. The (large) number of body-worn sensors/markers results in long setup times and can be obtrusive to the subjects.

Various studies have shown that using data-driven methods, a reduction in the number of sensors/markers for full-body motion capture is feasible by taking advantage of the inherent redundancy of human motion [15,16,17]. Chai and Hodgins have shown the potential of estimating full-body motion using only six retro-reflective markers, with a nearest neighbour search approach to map the low-dimensional marker input to full-body poses [18]. Note that poses are defined as the finite possible configurations of the body, i.e., a pose is a discrete sample of a motion sequence. However, their method, as most of the camera-based methods, is limited by the recording volume of the camera setup. To that end, Slyper and Hodgins used five body-worn accelerometers to estimate full-body motions in any environment, using a nearest neighbour approach as well [19]. The accelerometers were placed only on upper-body segments, which resulted in sub-optimal estimation performance of lower-body poses. This was further improved by Tautges et al. using a similar approach with four accelerometers placed on lower legs/arms [20]. These three methods include a cost function that weighs estimated poses in the past and present, which resulted in time coherent (plausible) output poses, since physically impossible large segment accelerations were smoothed.

All these approaches share the same “lazy learning” philosopy [21], since they don’t learn a universal model to estimate full-body poses, but rather rely on a database of pre-recorded motion to look up at runtime. This approach is computationally demanding and often results in a delay between the performed motion and the estimated full-body pose. The significance of that delay depends on the application, e.g., virtual reality requires minimal delays as it can lead to motion sickness [22,23], while providing feedback on gait analysis can be safely done with larger delays [6]. Opposed to lazy learning approaches, computation times can be reduced by using an eager learning approach (where a model is learnt and used at runtime), resulting in typically smaller delays. To that end, a shallow neural network was shown to estimate full-body poses from only five IMUs with comparable accuracy to lazy learning approaches [24]. However, estimated poses were not consistent over time, since time relations were not explicitly taken into account. A short-term movement prediction can accurately be made given characteristics of the dynamic system at hand [25]. This has been shown by Von Marcard et al. with their optimization framework that uses data of six IMUs to estimate accurate time coherent full-body motion [26]. However, this approach cannot be applied in real-time, since a long data sequence is required for optimal performance.

Time coherency and real-time are both requirements for various applications that use full-body motions. Deep learning has the potential to provide time coherent real-time full-body pose estimates as shown by the increasing use in estimating human motions from video. For example, Fragkiadaki et al. used long short-term memory (LSTM) units in their recurrent neural network (RNN) architecture to estimate full-body kinematics from colour videos [27]. Additionally, three-dimensional convolutional networks have been shown to be an effective network architecture for human activity recognition from videos [28]. Furthermore, optical motion capture can be complemented by inertial sensors and an LSTM architecture to improve visual tracking in the case of occlusions [29]. However, only bidirectional LSTM (bi-LSTM) units (to exploit information from both past and future) have been shown to accurately estimate full-body motion using the data of 6 IMUs. In this manner, time coherent (semi-)real-time output poses were achieved using an on-body measurement system. This approach was shown to result in the best performance by providing sequences that include past, current, and future frames as input; hence, output was delayed depending on the number of future frames required. However, such a deep learning architecture requires sufficient computational resources and a large dataset for training/evaluating.

In summary, estimating (real-time) full-body human motions from a minimal sensor setup can be achieved with good accuracy using offline methods (when a large motion sequence is available) or using deep learning at the expense of large datasets and computational resources. Our hypothesis is that similar results can be achieved by using a shallow learning approach. This resulted in the following research question: “What is the performance of a shallow approach, compared to a deep learning one, for estimating time coherent full-body poses using only five inertial sensors?” For the shallow learning approach, we propose a stacked input neural network (SINN) approach that requires smaller datasets and less computing power, which can result in suitability for real-time applications. This approach was based on earlier work of the authors [24], which showed good performance, but estimation of full-body pose at any given time only considered inputs at that instance, but not in the past. It therefore did not consider the inherent dynamics of the body that relate poses over time. In the current work, we developed a novel way of considering time dependencies in a shallow artificial neural network (ANN), namely, by moving complexity out of the (deep) network into a stacked input vector, which contains past and future information. The SINN approach was compared to a deep learning approach (with recurrent units) based on [30] (the current state-of-the-art for estimating full-body poses from a minimal set of inertial sensors ), which is referred to as a recurrent neural network (RNN) for simplicity. It was chosen to use inertial sensors as input, since this allows for a wearable motion capture solution that does not require external infrastructure. Furthermore, it has been shown that differences in joint angles between optical and inertial motion capture are small [31,32,33]. To understand the performance of both the SINN and RNN approaches, three aspects are analysed in more detail, namely: configuration of the stacked input (e.g., number of past/future poses and time intervals), the inclusion of acceleration input information, and the computational cost (for training and evaluating).

## 2. Methods

### 2.1. Movement Dataset

The dataset contains a wide variety of movements performed by six subjects, as described in Table 1. Approximately 25 minutes of motion capture data was collected for each subject. Xsens MVN (Xsens Technologies B.V., Enschede, the Netherlands) was used for recording the subject’s movements with 17 IMUs placed on (rigid) body segments at 240 Hz. Subjects performed a calibration pose to determine the sensor orientation with respect to the body, such that the biomechanical model of MVN Studio 4.2.1 (Xsens Technologies B.V., Enschede, the Netherlands) provides orientation of 23 body segments. The sampling frequency of 240 Hz resulted in nearly identical adjacent (in time) poses, and for most body parts, significant motion information lies well below 240 Hz. Therefore, the data were down-sampled by a factor of four (to 60 Hz), resulting in a dataset of approximately half million poses.

### 2.2. Input Features

**Figure 1:**
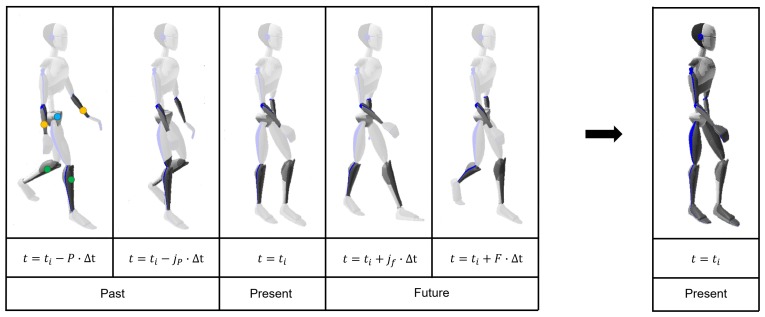
A sequence of inputs (lower arm (orange circle) /leg (green circle) orientations relative to the pelvis (blue circle) is used to estimate a single output pose (at time *i*). Size of the input sequence can vary by the number of past (*P*) and future (*F*) poses that are taken into account and the distance in time (Δt) between the different inputs. Here, $j_{p}$ and $j_{f}$ are used as counters for the past/future poses in time, which have a maximum value of P and F (in this example, P=2 and F=2), respectively. In other words, $j_{p}=\left\{1,...,P\right\}$ and $j_{f}=\left\{1,...,F\right\}$, and Δt is defined as $I/f_{s}$ with *I* as the sample interval and $f_{s}$ as the sampling frequency.

The recorded movement database contains orientations of 23 segments. Based on previous work in estimating full-body poses from a minimal body-worn sensor set [4,20,24,26,30], it was chosen to use the orientation of one segment for each limb as input features. Consistent with these works, the lower legs/arms and pelvis were selected as input segments (as highlighted in Figure 1), because they were positioned towards the ends of extremities. Orientation of these segments as provided by Xsens MVN was used as input, while the remaining segment orientations were used for the corresponding output. The main reason for reducing the dimensionality is to take advantage of the fact that human body poses are extremely redundant if global body orientation is considered [15,16,17]. However, if orientations are expressed with respect to the body (e.g., pelvis), this dimensionality is further decreased. In this manner, the input/output space is reduced by relating all input/output orientations to the pelvis orientation (marked by the blue circle in Figure 1). The choice of the pelvis as a reference segment is motivated by its central location with respect to the different limbs.

It was chosen to train independent SINN and RNN to estimate upper- and lower-body poses using orientations (and accelerations) of two body segments. This was based on the limited dataset and difficulties with learning such complex relations for a shallow network, which was successfully applied in a previous work of the authors [24]. In other words, the lower arm orientations (relative to the pelvis, marked by the orange circles in Figure 1) are provided to a trained network to estimate the upper-body segment orientations (12 segments), and the lower leg orientations (relative to the pelvis, marked by the green circles in Figure 1) are input to a second trained network to estimate the lower-body segment orientations (6 segments).

Quaternions were used to represent orientations in the dataset (directly obtained from Xsens MVN), since this representation was shown to be fitting for training an ANN to map a reduced set of sensors to a full-body pose, after normalizing the output to obtain proper unit quaternions [24,34]. Furthermore, in this manner, the input dimensions are smaller compared to rotation matrices, and they do not suffer from gimbal lock issues.

**Figure 2:**
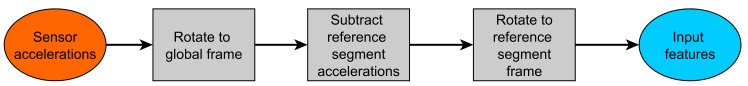
Processing of the measured sensor accelerations to be suitable input to the recurrent neural network (RNN) and stacked input neural network (SINN).

The IMUs measure 3D acceleration and angular velocity, of which the acceleration can be used as additional input to the network, since it provides information about the linear movement and segment inclination, which could therefore result in better time coherency between output poses. This hypothesis was tested by training additional SINN and RNN (including acceleration features) and evaluating differences in performance compared to using no acceleration features. Accelerations are measured in the sensor frame, i.e., to compare different sensor accelerations, a transformation is required, as shown in Figure 2. The first step is rotating individual sensor accelerations to a common global frame, which is achieved using the orientation of those sensors (which are expressed in an identical global reference frame). The pelvis acceleration is then subtracted from the lower legs/arms such that a relative acceleration is obtained, which also removes the gravitational acceleration from the resulting relative measure. Rotating that outcome to the pelvis orientation results in acceleration features that are relative to the reference segment and are not affected by the orientation of the body w.r.t. the world.

### 2.3. Stacked Inputs

The SINN was trained to map a sequence of inputs to one single full-body pose (i.e., input $x=\left[\begin{array}{ccccc} t_{i}-P\cdot \Delta t & t_{i}-j_{P}\cdot \Delta t & t_{i} & t_{i}+j_{f}\cdot \Delta t & t_{i}+F\cdot \Delta t \end{array}\right]$), as depicted in Figure 1. Δt is the time between different poses, which is defined as $\Delta t=I/f_{s}$, where *I* defines sample interval and $f_{s}$ the sample frequency (60 Hz). $j_{P}$ is a counter for the past poses (*P*), while $j_{F}$ is a counter for the future (*F*) poses that are taken into account. Additionally, acceleration features can be appended to this input matrix if required.

By doing this, we want to prove that the proposed SINN is able to “learn” time coherency even when past and future sensor data are stacked into the same input vector. This approach allows for various options for the number of poses over time that are considered (SIL=P+1+F, stacked input length) and the sample interval (*I*) between those adjacent poses. The optimal configuration depends on the requirements of the application, e.g., real-time applications, required accuracy and movement types. Figure 1 shows an example of a gait sequence using P=2, F=2 and I=8 (with Δt=1/60 s); therefore the length of the shown sequence is 5 samples that span approximately 0.55 seconds.

### 2.4. Network Architecture

**Figure 3:**
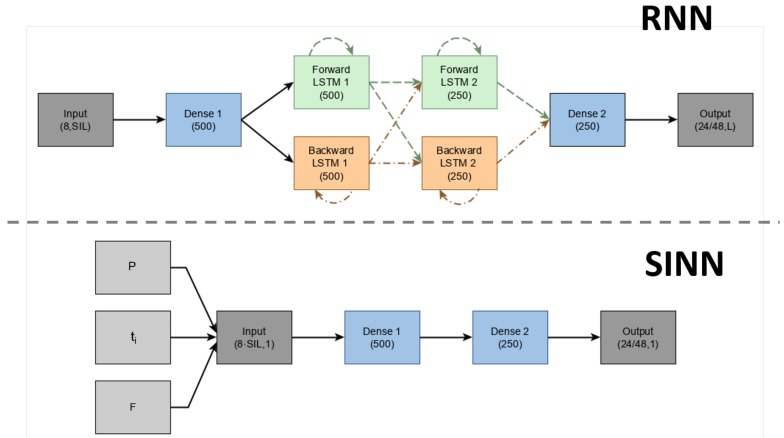
The implemented network architectures for the deep (RNN) and shallow (SINN) learning approaches. Different networks were trained for estimating upper/lower-body poses, which resulted in 8 inputs (2 segment orientations, represented by quaternions, relative to the pelvis) times SIL (stacked input length) poses for both SINN and RNN. Furthermore, a different number of outputs was obtained from the separate networks, namely, 24 for the lower body and 48 for the upper body. Input to the SINN is stacked with adjacent poses from past (P), current ($t_{i}$) and future (F) time samples, resulting in a total of L samples that are taken into account. The same sequence can be provided as an input matrix to the RNN, which produces a sequence as output (and the relevant pose can be used). The different types of hidden layers are shown by the various colours with the corresponding number of neurons shown in brackets.

Figure 3 depicts the network architectures for the deep (RNN) and shallow (SINN) learning approaches. The implemented RNN was inspired by the work of Huang et al. using bi-LSTM layers [30,35,36]. The network architecture of the RNN allows for recurrency and hence no input stacking is required. However, due to the bidirectional units in the networks, better qualitative and quantitative results can be obtained by processing a sequence of inputs (which is implemented as a sliding window) [30]. Therefore, both the RNN and SINN will be trained/evaluated using identical input sequences (in both length and configuration) obtained from our collected dataset. The difference is that the RNN gets a matrix of size (8,SIL) as input, while this is stacked to (8·SIL,1) for the SINN.

MATLAB R2018b (MathWorks, Inc., Natick, MA, USA) was used to implement both the SINN and RNN approaches. The following training parameters for the bi-LSTM network were the same as in [30], namely, using an Adam optimizer [37], identical learning rates and dropout [30]. The number of neurons per layer and number of hidden layers was chosen based on a previous work of the authors and was validated by comparing various network sizes. The number of hidden layers for the RNN approach was based on the work of Huang et al. [30]. Separate RNNs were trained for the upper- and lower-body movements due to the limited dataset and to provide a fair comparison with the proposed shallow approach (SINN).

Both the RNN and SINN were trained using a subject-wise six-fold cross-evaluation (5 subjects for training and 1 for evaluating). This evaluation approach provides information about the generalization performance of the trained networks over the subjects, as different subjects are in the training/test set for each evaluation [38,39].

### 2.5. Performance Evaluation

As commonly done in similar works, the accuracy of full-body poses is evaluated using either Euclidean joint distance [24,27] or joint angle errors [18,26]. The application largely determines the evaluation metric of interest. For conciseness, joint position errors are reported in the current paper, as these were shown to be related to how pose similarity is perceived by humans [40]. This was evaluated by calculating the Euclidean norm between the full-body joint positions obtained from Xsens MVN and the estimated joint positions from the SINN/RNN approaches. These 23 joint position errors were then averaged to obtain a mean error value for each pose.

Furthermore, a previous work of the authors has shown that jitter was present in the outcome pose sequences. It was chosen to quantify jitter by calculating jerk (the third derivative of the joint positions), since this provides insight in the smoothness error [41]. This was evaluated by calculating the Euclidean norm between the ground truth (Xsens MVN) and estimated jerk (SINN/RNN approaches).

Real-time applications require a delay that is not larger than the threshold that results in motion sickness [22,23]. However, this threshold is individual: a delay of 100 ms might be acceptable for some people, while others might not cope well with delays larger than 20 ms in virtual reality applications [42]. Therefore, the processing time of both the SINN and RNN approaches was evaluated on a notebook (Lenovo ThinkPad W540, Beijing, China) (CPU i7-4710MQ @ 2.50 GHz, 8 GB RAM, NVIDIA Quadro K1100M, Santa Clara, CA, USA), which is representative of equipment that can be used for a real-time application. As the number of future poses impact the additional delay between the measured movements and the estimated full-body poses, this parameter will be regarded. Furthermore, the difference in training time between both approaches was evaluated on a high-end machine (equipped with one NVIDIA GTX Titan X (Pascal 12 GB)), since training requires more computational resources.

## 3. Results

In this section, we explore the impact of different time window configurations on the SINN/RNN performance (Section 3.1). Furthermore, the addition of accelerometer data in the input is investigated (Section 3.2) and the computational cost of both approaches is compared (Section 3.3). Additionally, videos of the obtained output have been included as Supplementary Materials.

### 3.1. Time Window Configurations

The considered dataset consists of three activity types, namely, gait, sports and ADL. The dynamics of different activities can vary substantially, which could result in variation of the optimal time windows (length, configuration and spacing). To that end, mean (over six subjects) joint position and jerk errors are presented for a representative trial within each of those activity classes.

**Figure 4:**
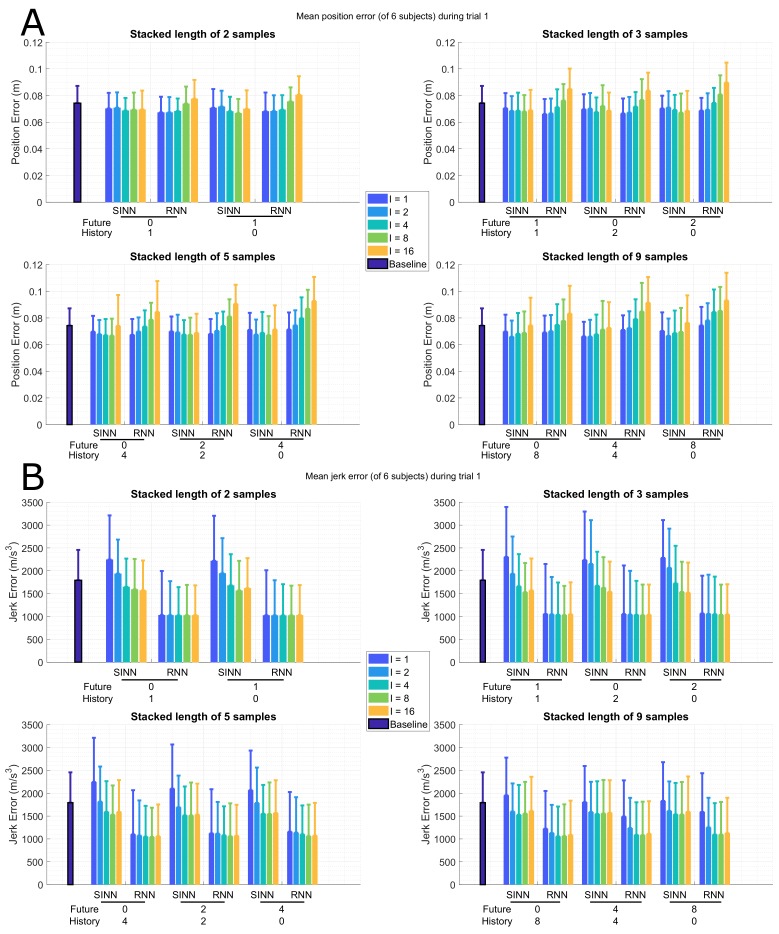
Bar plots of the full-body mean (of 6 subjects) joint position (**A**) and jerk (**B**) error for the shallow (SINN, left bars for each configuration) and deep (RNN, right bars for each configuration) learning approaches during a gait trial (1 in Table 1), standard deviation over the various subjects is displayed by whiskers. The different time windows are presented on the *x*-axis, where the number of past (*P*) and future (*F*) poses are shown. The interval (*I*) between input poses are marked by the different colours, where the number of samples between input poses is shown. For comparison, the mean joint position error (**A**) for using only the current pose as input (SINN approach) is 0.07 (±0.01) m. For comparison, the baseline using only the current pose as input) mean joint position (**A**) and jerk (**B**) errors are shown as the dark blue bars on the left.

Figure 4A shows the mean joint position errors for a gait trial, namely, for trial 1 as described in Table 1. With increasing distance (*I*) between included samples, the mean joint position error shows an increase for all different SIL with the RNN approach. This trend can also be seen for the SINN approach; however, the absolute error increase is smaller. Furthermore, the standard deviation of all mean joint position errors (for both approaches) is of similar small size (~0.01 m), indicating good generalization over different subjects. The impact of using information from past or future (stacked input configuration) is shown to be minimal, while the SIL (number of samples) has a larger effect.

Figure 4B shows the mean (and standard deviation) joint jerk errors for the different time window configurations of trial 1, as described in Table 1. The RNN shows smaller jerk errors than the SINN approach, which was to be expected since the RNN explicitly takes into account time coherency of the different poses in the input sequence. Furthermore, for increasing intervals (*I*), smaller joint jerk errors can be seen in the SINN approach, while this effect is only shown for the larger SIL (of 5/9 samples) of the RNN approach. Compared to using only a single pose as input to the SINN, a decrease in joint jerk error is observed when the SIL is larger (i.e., more time information is taken into account). Furthermore, the stacked input configuration has a smaller effect on performance than the SIL, similar to what was observed for the joint position errors.

**Figure 5:**
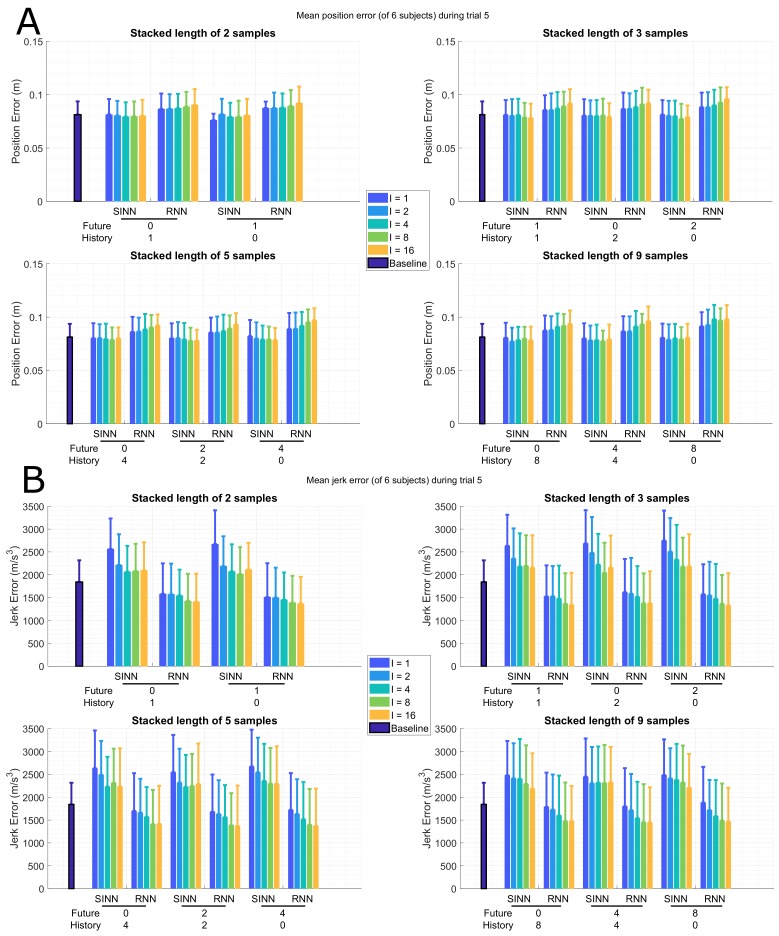
Bar plots of the full-body mean (of 6 subjects) joint position (**A**) and jerk (**B**) error for the shallow (SINN, left bars for each configuration ) and deep (RNN, right bars for each configuration ) learning approaches during an ADL trial (5 in Table 1), standard deviation over the various subjects is displayed by whiskers. The different time windows are presented on the *x*-axis, where the number of past (*P*) and future (*F*) poses are shown. The interval (*I*) between input poses are marked by the different colours, where the number of samples between input poses is shown. For comparison, the mean joint position error (**A**) for using only the current pose as input (SINN approach) is 0.08 (±0.01) m. For comparison, the mean joint jerk error (**B**) for using only the current pose as input (SINN approach) is 1.8 (±0.5) $\times 10^{3}$ m/s^3^.

Overall increase in joint position errors for sports activities compared to gait is observed for both approaches (**SINN**: median(gait)=0.069 m and median(sports)=0.079 m; **RNN**: median(gait)=0.076 m and median(sports)=0.089 m) in a more dynamic activity, as can be seen for trial 5 in Figure 5A, which includes sport related tasks (as described in Table 1). For the SINN approach, the interval (*I*) shows a smaller effect on the joint position error than for the RNN approach. A decrease in the joint position errors for both approaches can be seen when more information is taken into account (larger SIL).

As was to be expected for a more dynamic trial, the joint jerk error is larger for sports activities than for a gait trial (**SINN**: median(gait)=1609 m/s${}^{3}$ and median(sports)=2309 m/s${}^{3}$; **RNN**: median(gait)=1056 m/s${}^{3}$ and median(sports)=1515 m/s${}^{3}$), shown in Figure 5B. Similar to the gait trial, a decrease in joint jerk error is observed for large *I*; however, this effect decreases for larger SILs. Furthermore, both approaches show minimal differences in performance for various stacked input configurations (*P*/*F*) with a fixed SIL.

**Figure 6:**
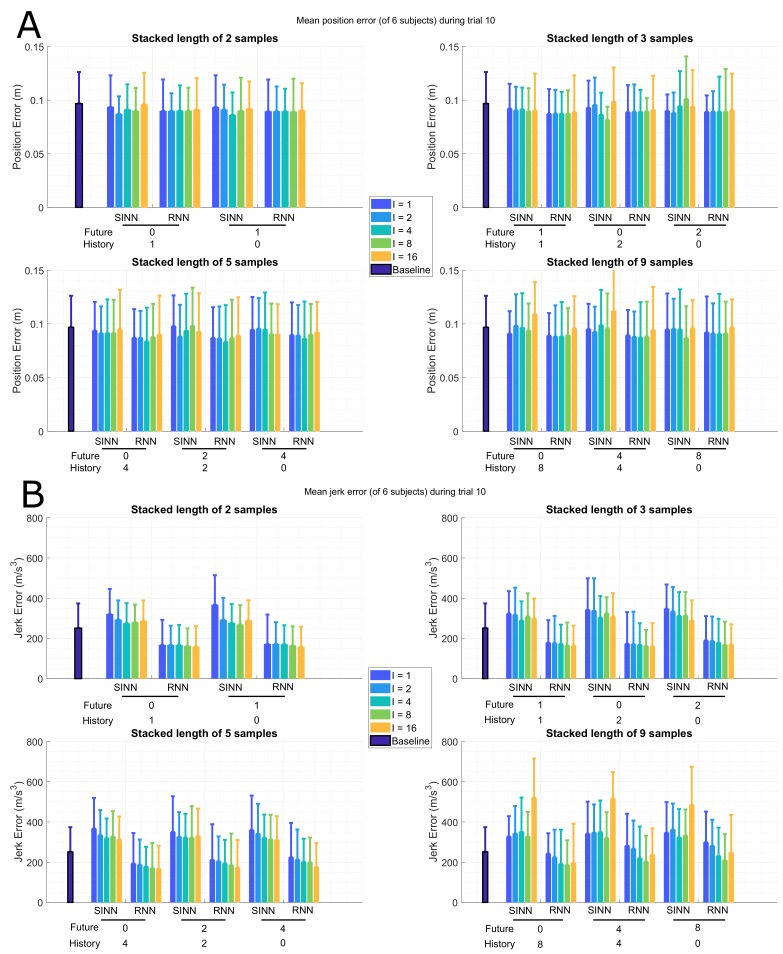
Bar plots of the full-body mean (of 6 subjects) joint position (**A**) and jerk (**B**) error for the shallow (SINN, left bars for each configuration ) and deep (RNN, right bars for each configuration) learning approaches during a sports trial (10 in Table 1), standard deviation over the various subjects is displayed by whiskers. The different time windows are presented on the *x*-axis, where the number of past (*P*) and future (*F*) poses are shown. The interval (*I*) between input poses are marked by the different colours, where the number of samples between input poses is shown. For comparison, the baseline (L**A**) for using only the current pose as input mean joint position (**A**) and jerk (**B**) errors are shown as the dark blue bars on the left.

Figure 6A,B shows, respectively, the joint position and jerk errors during an ADL (as described in Table 1). Similar to the previous two activities, an error decrease is observed for larger SIL, but no large differences for the various configurations (*P*/*F*) are observed in this activity either. A decrease in performance is shown for larger *I* but is not consistent between both approaches.

Even though the dynamics are different between the three activities, it can be seen that small *I* results in a joint position error increase (shown in Figure 4A, Figure 5A and Figure 6A), as the similarity between those poses is too high, and therefore, the individual poses contain minimal additional information, while large *I* results in an increase of the joint jerk errors (shown in Figure 4B, Figure 5B and Figure 6B), since dependency between poses decreases at larger time intervals. In other words, there is an optimal interval *I*, which depends on the specific dynamic nature of the activity. This optimum can therefore be found around I=2 and I=4, since this results in smaller joint position and jerk errors on average over the various subjects and activities.

Configuration of the input vector (past/future) shows smaller effects on the joint position/jerk errors than the SIL. This effectively means that a longer sequence of inputs is more beneficial than changing the configuration of those inputs, e.g., including future information at the expense of past information. However, a marginal error decrease was observed when future information was included. Hence, for real-time applications a larger SIL can be sufficient, while for applications that require higher accuracy, it can be valuable to include future information.

For sake of simplicity, in the remainder of the paper, we will use I=2, P=2 and F=2 as this configuration resulted in an acceptable trade-off of accuracy and possibilities for real-time applications.

### 3.2. Including Sensor Acceleration Features

**Figure 7:**
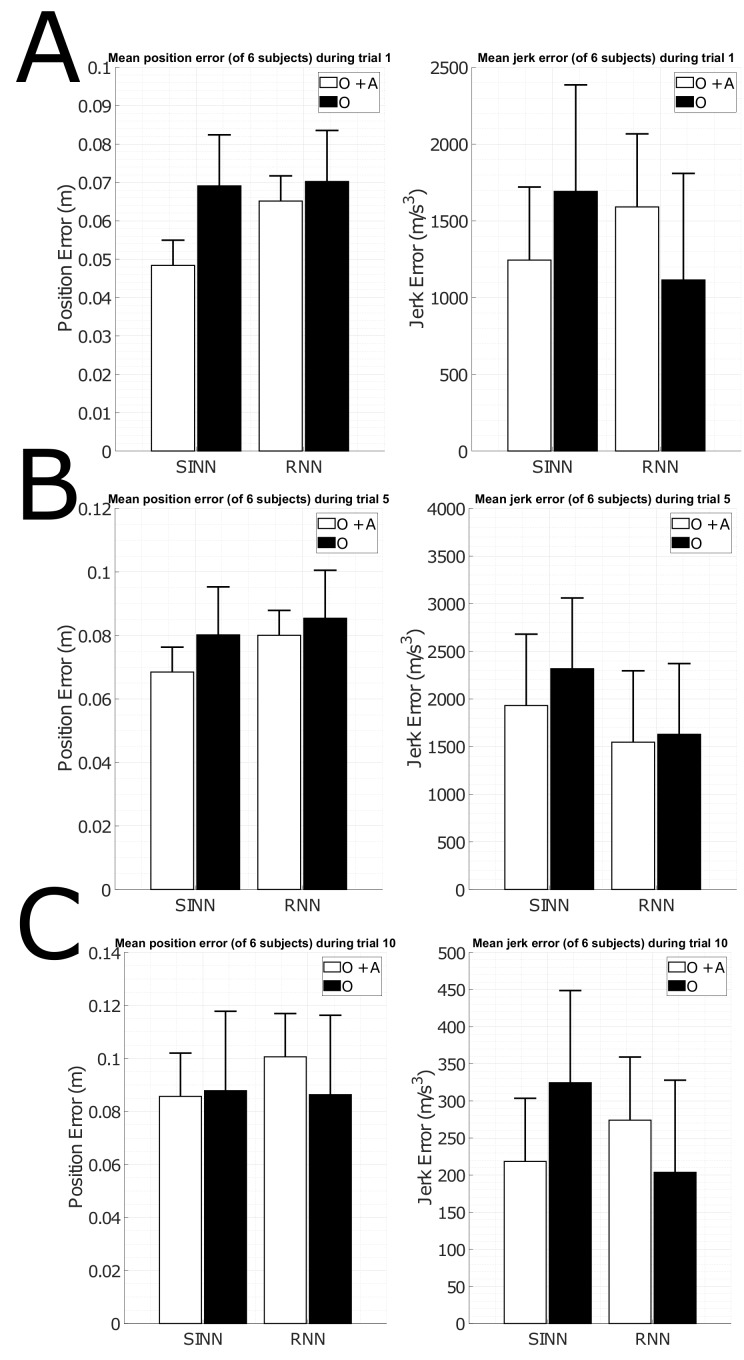
Bar plots of the mean (of 6 subjects) joint position and jerk error for the shallow (SINN) and deep (RNN) learning approaches (left and right, respectively) using orientation features (**O** in black) and including accelerations (**O + A** in white). Three different types of activities are shown namely: gait (**A**), sports (**B**) and ADL (**C**). Standard deviation over the various subjects is displayed by black whiskers. These results were obtained using the following parameters: I=2, H=2 and F=2.

Figure 7 shows the mean joint position/jerk errors for three different activities, namely, gait, sports and ADL, using orientation features (as shown in Section 3.1) and including accelerations. The gait and sport trials show a decrease in joint position error for both the SINN and RNN approaches when acceleration features are included compared to only orientation features. This is not the case for the ADL trial, which could possibly be the result of decreased dynamics in ADL tasks, hence acceleration information could provide less additional knowledge. The joint jerk errors are smaller when acceleration features are included for the SINN approach. This is only observed for the sports trial in the RNN approach. This indicates that including acceleration features improves full-body pose estimation, but it can be at the expense of smoothness of the output.

### 3.3. Delay Assessment

A delay is already introduced by using future sensor information as input to both the SINN and RNN approaches. The chosen input sequence configuration (2 future poses) results in a delay of 67 ms using MATLAB R2018b (MathWorks, Inc., Natick, MA, USA). In Table 2, the training and calculation times are presented. It should be noted that these results are obtained from training/evaluating a SINN and RNN for both the upper and lower body, which requires double the amount of training time, but could be performed in parallel. It can be seen that a shallow network, as expected, is faster to train and evaluate. However, the RNN approach does not require a new training cycle when experimenting with the input sequence configuration, e.g., when more past/future frames should be taken into account.

## 4. Discussion

In this work, we have shown that using either a shallow (SINN) or deep (RNN) learning approach for estimating full-body poses using only five IMUs placed on the lower legs/arms and the pelvis results in similarly accurate outcomes.

A limitation of this work is that input to both learning approaches was the segment orientation from Xsens MVN, i.e., the calibrated sensor to segment pose data were based on the full-body approach [10]. This full-body approach benefits from assumptions based on dynamics of a human body, which is not the case for a single sensor. However, the orientation accuracy of an IMU is within 0.5 degrees [43]. Furthermore, an application with only five inertial sensors would require a sensor to segment calibration, such as the static neutral pose proposed by Huang et al [30]. A misalignment of the sensors with respect to the calibration pose could decrease the accuracy of the estimated full-body poses; however, it has been shown that sensor noise has a minimal impact on the performance of such a trained neural network [24]. The SINN/RNN approaches could also be trained to handle inputs with noise, such as was done for optical pose estimation [44].

The input and reference data consisted of inertial motion capture data. In this manner, no external infrastructure is required for data collection. The accuracy of inertial motion capture is comparable to that of optical systems when looking at joint angles [31,32,33]. However, the use of optical motion capture data provides interesting opportunities for enlarging the training dataset, since such data are publicly available [45,46,47]. Our proposed SINN approach could benefit from such datasets if they were recorded with sufficient optical markers, since it requires three-dimensional orientations as in/output. The relatively small dataset used in this work allowed for a fair comparison with the previous work of the authors [24] and demonstrated that a shallow approach trained on such a dataset can estimate full-body poses with good accuracy. It should be noted that a shallow network requires significantly fewer parameters to be trained (the SINN approach has approximately 25 times fewer trained parameters compared to the RNN approach), which impacts the minimal required training dataset size.

It was chosen to use I=2, P=2 and F=2 as an input configuration to evaluate the addition of acceleration features, due to it being an acceptable trade-off between accuracy and delay. However, this choice largely depends on the application and dataset as differences were also observed for the various activities. Variations in the optimal settings for the various activities could be the effect of differences in the involved dynamics. However, more insight in this relation is required, which could reduce the search for optimal settings for specific applications. Therefore, this setting is not a final recommendation, but the presented results can provide a direction for specific applications.

The gait trial was estimated with the smallest position error, which was to be expected due to the repetitive and cyclic nature of the activity. The largest errors can be found in the activities that are less cyclical, such as ADL. A trend observed for all activities is that the position error decreases when more information was used as input (SIL). This effect was larger than changing the input vector configuration (number of poses from past/future). However, including information about the future resulted in a decrease of joint jerk errors, i.e., more smooth outcomes. This was to be expected, since interpolating is a less error-prone task than extrapolating [48]. Joint jerk errors were further improved by including acceleration information as input to both the RNN and SINN approaches, as can be seen from the improved joint position errors for all trials.

These observations of use of a RNN approach for estimating full-body movements using a minimal sensor set are consistent with the findings presented in [30]. Since a different dataset for training/testing was used in their work, which could indicate that these effects are not dataset-dependent. Joint position accuracy reported by Huang et al. was 6.49 cm on average (for their RNN approach) compared to the 7.33 cm (mean of Figure 7) reported in this work. A larger joint position error can be the result of a smaller dataset and/or of training SINN/RNN for the upper/lower body separately. The mean joint position error for the proposed SINN approach is 6.23 cm. This error is smaller than both the reported error of the RNN approach in this work and that of Huang et al. However, these differences are small in magnitude, namely, approximately 1 cm. This indicates that the proposed SINN approach can provide an alternative for estimating full-body poses using only five IMUs for the RNN approach, requiring less computation power and training data.

Large jumps in outcome poses that were observed using a snapshot approach [24] have been reduced by using a stacked input vector, as can also be seen from the supplementary material. Furthermore, the mean joint position error is approximately 2 cm smaller than the one reported in [24]. In addition, the joint position error of individual joints showed a similar distribution to that observed in previous work. This was to be expected due to the kinematic chain that is evaluated, which effectively accumulates joint position errors from the proximal joints to the more distal joints. Joint jerk errors improved compared to previous work, which indicates that time coherency between outcome poses can be improved by stacking poses in the input vector. However, RNN results show smaller joint jerk errors than the SINN results, which shows that more time coherent outcomes can be obtained by explicit recurrent modelling.

The combined delay from the chosen configuration and reported computation time is 72 ms for the SINN approach and 117 ms for the RNN approach using a MATLAB implementation. These reported computation times are only indicative, since shorter delays are expected for a C++/firmware implementation. The SINN approach can be used to estimate full-body poses within acceptable delay boundaries (20–100 ms, according to [42]); however, the delay is close to the upper boundary. This can be improved by using less future information at the expense of accuracy. The RNN approach delay can be improved in a similar fashion or by using higher computational power. Alternatively, cloud computing could provide a more powerful computing environment without requiring such powerful equipment on site [49]. However, feasibility of such a solution for real-time pose estimation largely depends on the available internet speed.

Results presented in this work allow for decreasing the number of sensors/markers in full-body motion capture. While this approach is not tailored to any specific application, it was shown that cyclical and repetitive motions are estimated with the highest accuracy. Therefore, this approach has the largest potential to be applied to activities with these characteristics, e.g., biomechanical analysis of running [4], providing biofeedback to patients [6] or industry applications [50]. However, applications with less cyclical/repetitive motions may require more fine-tuning effort to reach the required level of accuracy.

### Future Work

The results in this work were obtained by training a shallow/deep learning approach on a relatively small dataset. The effect of dataset size on performance of the proposed approaches remain unclear, and would require further analysis. The increased number of publicly available datasets [45,46,47] could provide opportunities for such an analysis.

The results in this work were based on inputs from the full-body motion capture output of Xsens MVN, while using orientations of five single IMUs directly might result in a decreased performance for estimating full-body poses. Therefore, additional research is required to evaluate the use of orientation and acceleration input of five single IMUs.

Performance of the proposed approaches varies with dynamics of the evaluated activities. A concept that could potentially improve this effect is to apply an adaptive time window based on the acceleration data, e.g., longer input sequences for low dynamic activities and the opposite for high dynamic activities. This concept can be applied to the RNN approach directly, while the SINN approach would require various trained networks (with different settings), which can then be chosen to use at run-time depending on the dynamics of the activity.

The current analysis was performed using MATLAB; for development of a specific application, it should be preferred to use a different programming environment, such as Python or C++. This would likely also result in improved evaluation delays; however, the presented delay results can provide a benchmark.

## 5. Conclusions

The goal of this work was to evaluate the performance of estimating full-body poses using only five IMUs with a shallow (SINN) compared to a deep (RNN) learning approach. It has been shown that similar joint position accuracy (SINN ≈ 6 cm and RNN ≈ 7 cm) was achieved for both approaches with the considered dataset. However, the RNN approach results in smaller joint jerk errors, which is possibly the result of the explicit recurrency of the network. Furthermore, the SINN approach estimates poses with smaller delays, which allows for real-time applications. However, a SINN approach provides no flexibility to change the size and configuration of the stacked input vector at run-time. Therefore, choosing either approach would depend on several factors, namely, available computing power, dataset size, and/or real-time requirements.

## Figures and Tables

**Table 1:** A description of trials in the experimental protocol (each trial was performed three times by all six subjects). ADL = Activity of daily living, L = left and R = right [24].

	Trial	Short Description
Gait	1	Walk 10 m, walk 10 m, jog 10 m and sprint 10 m.
	2	Walk with a glass of water (dominant hand, non-dominant hand and in both hands)
	3	Walk 10 m, walk slowly 10 m, walk backwards 10 m, side-step six steps (L/R).
Sport	4	Lunges L/R (4×), squats (4×), jumping jacks (4×).
	5	Two-legged jumps (4×), hops L/R (4×), run and jump L/R (2×), jump up (4×).
	6	Sit-ups (5×) and side side-ups L/R (3×).
	7	Kick a ball against the wall L/R (3×).
	8	Throwing a ball against the wall L/R (3×).
	9	Crawling six steps.
ADL	10	Take a magazine, put it on the table, get seated, read a magazine, stand up and put it away.
	11	Take a tray with cups, walk with the tray, put it on the floor, stand up, pick it up.
	12	Take a glass, fill it with water and drink it in a chair.
	13	Put on a coat and take it off.
	14	Comb hair, scratch back, touch toes, rotate arms around shoulder back- and forward.
	15	Kneel down and tie shoelaces (L/R).
	16	Ascend and descend stairs.

**Table 2:** Training of both approaches was done using a machine equipped with a single NVIDIA GTX Titan X (Pascal 12 GB) with MATLAB R2018b. Testing was done on a notebook, namely, a Lenovo ThinkPad W540 (CPU i7-4710MQ @ 2.50 GHz, 8 GB RAM, NVIDIA Quadro K1100M) with MATLAB R2018b.

Approach	Training Time (Hours)	Evaluation (ms/Sample)
RNN	~6	~50
SINN	~1	~5

## Supplementary Materials

The following are available online at https://www.mdpi.com/1424-8220/19/17/3716/s1 (accessed on 28 August 2019). Video S1: Shallow versus Deep Learning.

## Abbreviations

The following abbreviations are used in this manuscript:

ADL	Activities of Daily Living
ANN	Artificial Neural Network
IMU	Inertial Measurement Unit
LSTM	Long Short-Term Memory
RNN	Recurrent Neural Network
SIL	Stacked Input Length
SINN	Stacked Input Neural Network
